# Does CEO social capital affect corporate ESG performance?

**DOI:** 10.1371/journal.pone.0300211

**Published:** 2024-11-05

**Authors:** Wenyi Wu, Zhaoping Tian, Guangzhi Wang

**Affiliations:** Zhe Jiang Guangsha Vocational And Technical University Of Construction, Jinhua, Zhejiang, China; University of Baltistan, PAKISTAN

## Abstract

This paper empirically examines the relationship between CEO (Chief Executive Officer) social capital and corporate ESG (Environment, Social and Governance) performance. Using a sample of A-share listed companies in Shanghai and Shenzhen from 2010 to 2020, the paper finds a negative correlation between CEO social capital and corporate ESG performance. In addition, we also consider how the firm’s market trading activity and CEO duality moderates the impact of CEO social capital on firms’ ESG, and both are concluded to be positively moderated. Upon further research, we also find that (1) the positive contribution of CEO’s social capital to firms’ ESG performance is more significant in state-owned enterprises. (2) The negative facilitating effect of CEO’s social capital on corporate ESG performance is more significant in large-scale enterprises. (3) ESG practices lead to the loss of shareholder wealth, resulting in the reduction of corporate value. The results of the study deepen the knowledge of academics and practitioners about the value-creating function of CEO social capital, and provide empirical evidence for listed companies to pay attention to and make use of CEO social capital to enhance their corporate social responsibility commitment.

## 1. Introduction

In recent years, the ESG system is gradually becoming a consensus and trend, and the demand for social responsibility arising from sustainable development is further reshaping the development concepts of various countries [1]. In China, listed companies have better disclosure of financial information, but the disclosure of ESG information, such as corporate governance and environmental protection, is obviously insufficient, which affects the market’s identification of enterprise-related risks. The reasons for this are, first, the lack of an authoritative consensus standard for ESG disclosure in the market, and second, the insufficient willingness of companies to disclose ESG information on their own initiative. In September 2020, President Xi Jinping announced at the 75th United Nations General Assembly that carbon dioxide emissions are striving to reach a peak by 2030. China will strive to achieve the goal of "carbon neutral" development by 2060. To realize this development concept, integrating environmental, social and governance factors into investment decisions is necessary to address global social issues at the micro level. Moreover, it is also an effective means of realizing China’s economic transformation and promoting high-quality economic development [2]. By the end of October 2021, a total of 19 public funds in China had signed the Principles for Responsible Investment, and the number of countable pan-ESG public funds amounted to 344, with a size of 549.2 billion yuan, and the total amount of green bond issuance was about 1,650 billion yuan. In recent years, China has been strengthening the ESG disclosure system for listed companies and encouraging listed companies to disclose information on environmental and social responsibility.

With the increased attention to corporate ESG performance from all walks of life, in academia, scholars have extensively explored the influencing factors of corporate ESG performance. The influencing factors of ESG include external factors, such as society [1], banks [3], government [4], investors [5], media [6], and other interested third party concerns. It also includes internal factors such as corporate values [7], management traits [8], and shareholder bias [9]. Management as one of the internal factors is the core layer that undertakes the execution of business decisions. His behavior will undoubtedly have a significant impact on business decisions and sustainable development strategies. According to the firm resource theory, a firm’s long-term competitive advantage stems from special resources that cannot be imitated by other firms, such as the CEO’s social resources. As an important position in corporate management, CEO personal traits may have an impact on corporate sustainability strategies [10]. We argue that CEOs inevitably engage in social interactions with internal and external stakeholders in their daily work as well as in their daily lives, which results in the formation of their personal social capital. CEO’s social capital as a complement to personal exogenous traits. It can significantly affect personal cognition and decision-making, and the potential resources behind social capital can bring more invisible resources and benefits to the firm [11]. Along this line, this paper argues that CEOs’ social capital has an impact on firms, including their ESG performance, both at the level of personal perceptions and at the level of social resources.

However, there is still relatively little academic research related to the relationship between executives’ traits and ESG performance. Previous studies of managers’ external characteristics in the literature [12–14] have focused on the explicit resources of executives, such as gender, education, etc., while there is a gap in the exploration of social capital. However, social capital is a special resource for executives that cannot be ignored and has an impact on their behavioural choices, which ultimately plays a role in corporate governance decisions [15, 16]. At the same time, the influence of CEOs’ social capital can vary across firms, and we need to further analyse the drivers and mechanisms by which executives’ social capital affects firms’ ESG performance.

To explore the above research gaps, we ask four questions. First, does CEO social capital affect corporate ESG performance? Second, does the nature of corporate ownership and size affect the relationship between CEO social capital and corporate ESG? Third, does CEO duality and the degree of active market trading moderate the impact of CEO social capital on corporate ESG? Fourth, does CEO social capital affect firm value? Therefore, this study examines the mechanism of the impact of CEO social capital on ESG performance and broadens the research framework of the impact of executive social capital on corporate governance. At the same time, we test the economic consequences of ESG performance under the influence of CEO social capital. Our study not only contributes to a better understanding of the drivers of CSR in academia and practice, but also provides a reference basis for the personal development choices of CEOs and the policy formulation of firms in hiring executives in practice.

The remaining paragraphs of the article are organized roughly as follows: the second part are literature review and hypothesis development; the third part is research design; the fourth part contains the main results and analyses; and the fifth part are research conclusions and practice implications.

## 2. Literature review and hypothesis development

### 2.1 Literature review

#### 2.1.1 Research on the economic consequences of executive social capital

The concept of social capital was first introduced by the French scholar Bourdieu, who defined it as "the aggregate of real or potential resources" [17]. Later, Nahapiet and Ghoshal further defined it as "the sum of explicit and implicit resources that are embedded in the network of social relationships and can be acquired and derived from it" [18]. That is, both firms and their executives have more or less connections with organizations or individuals outside the firm, such as with banks, government agencies, competitors, customers, suppliers, industry associations, alumni, etc., and such connections are accordingly transformed into social capital for firms and executives.

Thus, as a complement to the firm’s exogenous resources, scholars at home and abroad have tried to explore the economic consequences of the potential resource of social capital of the firm and its executives in corporate governance. On the one hand, executives’ social capital can bring some positive effects to firms, such as executives with financial backgrounds [19] and political backgrounds [20] help firms to lend more money and venture capital at lower interest rates [21], reduce firms’ cost of equity capital [22], significantly improve firms’ investment efficiency [23] and offsite M&A performance [24], and directly influence firms’ diversification strategies [25], increase firms’ risk-taking levels, and lead to increased firm value [26]. For innovative firms, executive social capital also helps innovative firms obtain valuable information needed for innovation [27]. On the other hand, executive social capital may also have some negative impact on the firm. For example, executives’ "trade association" tenure can lead to overinvestment [28], executives’ political social capital may have a "crowding-out effect" on firms’ R&D investment, and CEOs’ business social capital may inhibit green innovation [28]. Social capital of executives may have a "crowding out effect" on firms’ R&D investment, and CEOs’ business social capital may inhibit green innovation [29].

#### 2.1.2 Influences on ESG performance

ESG has gradually become a hot topic in both academia and industry over the last decade. By improving their ESG performance, firms can help avoid business risks [30], reduce financing costs [3], and improve investment efficiency [5]. Meanwhile, good ESG performance implies that firms are more focused on long-term value and sustainability [31]. As capital markets are gradually incorporating ESG scores into investment and financing considerations, many listed companies have begun to actively participate in ESG practices in order to gain the trust of investors and creditors [8]. The influencing factors of ESG performance may be as follows:(1) Market and governmental supervision: compared to the European market’s well-established system on ESG disclosure, Asian markets have also been actively introducing ESG-related regulations in recent years to provide investors with more standardized disclosures [4]. In order to achieve carbon neutrality by 2060, the Chinese government has given full play to its role in guiding corporate ESG practices, actively constructing a high-level socialist market economic system, exploring the establishment of a valuation system with Chinese characteristics, focusing on the maximization of economic benefits and social value, and focusing on long-term, sustainable value creation [32]. In addition, the Chinese government has carried out a series of policy support, and enterprises with ESG advantages can get system tilts including tax incentives, financial subsidies, and other policies to reduce the cost of corporate innovation and improve the level of innovation [33]. (2) Investor preferences: in addition to active government guidance, institutional and individual investors play an important role in influencing ESG investment practices, challenging companies’ ESG performance, and promoting ESG performance in the portfolios they manage [6]. In recent years, investors have begun to incorporate ESG into their thought processes [13]. Some studies have shown that ESG investors play a positive moderating role in the relationship between ESG performance and financial performance [12]. Michela Cordazzo’s study states that voluntary environmental disclosure is value-relevant and contributes to improved market functioning [7]. Broadstock find that high ESG investment portfolios typically outperform other ESG portfolios and that ESG performance mitigates financial risk during financial crises [34]. (3) Media monitoring and corporate social reputation: In the context of economic globalization, the media has a great deal of power and a certain degree of public influence, and the information it releases is likely to influence the decision-making judgment of consumers and investors. Resource base theory suggests that a good CSR reputation is an intangible resource that provides a sustainable competitive advantage and benefits firms through improved financial, investment and economic performance, increased employee productivity and easy access to financial resources [35]. Conversely, negative media coverage may adversely affect a firm’s reputational capital and send negative signals to investors [36]. In order to enhance social reputation or avoid the risk of scandal, firms may be pressured by the media to actively engage in ESG practices [37].

According to the existing literature, studies on the economic consequences of executive social capital either individual executives or the social capital of the executive team can bring more information and resources to the firm [16], reduce transaction costs, avoid business risks, influence business strategy as well as enhance business performance and firm value [38]. As for the ESG performance of enterprises, scholars’ current studies on its influencing factors are mostly from the external perspective of enterprises, such as market and government supervision, investor preferences, and factors such as media supervision and corporate social reputation, but rarely from the internal perspective of enterprises, and few scholars have considered the influence of executives on the ESG performance of enterprises. The CEO is an important member of the management of the company, and possesses a large amount of social capital. His social capital is also one of the firm’s intangible resources, which is likely to affect the firm’s ESG performance. Therefore, this paper investigates the relationship between the CEO’s social capital and ESG performance with important theoretical significance and practical value.

### 2.2 hypothesis development

#### 2.2.1 CEO social capital and ESG performance

Agency theory suggests that CEOs, as professional managers, usually consider their own interests when making decisions, causing CEOs to exhibit a risk-averse style when making decisions [7]. On the one hand, ESG practices inevitably imply the investment of certain costs and are characterized by large investment amounts as well as uncertain returns. When the enterprise’s resources are limited, the investment in ESG will certainly crowd out the investment in other projects such as technological innovation, which will inevitably increase the financial burden of the enterprise in the short term and affect the enterprise’s financial performance [39]. On the other hand, for CEOs, short-term business performance tends to be more intuitive proof of their decision-making correctness and professional competence than the delayed and uncertain benefits that ESG practices can bring, and it can also most directly satisfy stakeholders’ needs for maximizing corporate value [40]. If CEOs use their funds for environmental protection and social good, it will affect the CEO’s recent performance appraisal and personal evaluation because ESG activities increase costs, deplete the firm’s cash flow, and weaken the firm’s competitive advantage [41]. As a result, CEOs may care more about short-term financial performance and not focus on long-term sustainability, thus reducing or abandoning investment in ESG practices.

CEOs may not support the ESG development of their firms for self-interested motives. In contrast, CEOs with social capital are more likely to have the opportunity and the right to engage in "self-interested behavior" and are more likely to increase the risk of "self-interested behavior" going undetected due to their rich social connections. On the one hand, CEOs with social capital have more close ties with shareholders within the firm as well as with various outside stakeholders such as suppliers and customers [42], and such close ties tend to give CEOs greater voice and decision-making power in the firm. Shareholders may place unreasonable trust in the CEO because of their interactions with him and give him too much power over the internal management of the firm; major customers, as well as suppliers, may be pressured not to question the professional competence of the CEO in their business dealings because of their close interactions with him. The rights brought by social capital provide favorable conditions for CEOs to avoid long-term corporate investments such as ESG. On the other hand, CEOs with social capital form small groups with some other managers and stakeholders [43], and the imbalance in the monitoring mechanism between management may lead to a partial or total failure of the internal control system of the enterprise, and the risk of "self-interested behavior" going undetected increases. What is more, the management team forms a complicit relationship that jeopardizes the interests of the firm and the implementation of ESG. Based on this, this paper proposes hypothesis H1a:

**H1a:** CEO social capital has a negative effect on corporate ESG performance.

According to the Enterprise Resource Theory, the personal attributes of the CEO, as a decision maker and implementer, can have a direct and far-reaching impact on the growth and long-term sustainability of the organization [40]. The CEO’s rich social connections can extend to stakeholder groups such as customers, suppliers, competitors, partners, and governmental agencies. According to social capital theory, these connections that transcend firm boundaries can form the CEO’s personal social capital and provide real and potential resources for the CEO individually and for the firm, as well as influence the development and selection of the firm’s strategic decisions [44], including the firm’s ESG performance.

First, the personal traits of CEOs determine their cognitive abilities and values, and CEOs with more social capital may pay more attention to integrating stakeholders’ demands. They are more willing to seek corporate development from a long-term perspective, actively fulfill corporate social responsibility, endorse the concept of sustainable development, and promote ESG practices [45]. Second, CEOs’ rich social capital broadens the company’s financing channels, reduces the cost of capital, alleviates CEOs’ concerns about the financial burden caused by ESG inputs to the company, and provides environmental support for ESG practices [46]. Moreover, CEOs tend to be exposed to the top management of various companies or organizations, and thus their social networks are also invisible resources. When an unfavorable situation occurs, the strong social capital he possesses will also help him to solve the dilemma, which to some extent reduces the CEO’s concern when there is uncertainty in taking social responsibility decisions [47]. In addition, CEOs with social capital may be in a favorable position to obtain hot or critical information, and this informational advantage alleviates information asymmetry [48]. In the CEO’s social network, information about regulatory policies and regulations on corporate governance and environmental protection implemented by government departments, investors’ concerns about corporate ESG performance, how other managers in the same industry are actively promoting ESG practices, and the increasing environmental awareness of customers (consumers) can be quickly accessed by the CEO using his or her information vantage point. In the process of ESG practices, the key information acquired by CEOs can optimize long-term corporate sustainability strategies and better enhance corporate ESG performance [49]. In summary, this paper proposes hypothesis H1b:

**H1b:** CEO social capital has a positive effect on corporate ESG performance.

#### 2.2.2 Further study of CEO social capital and corporate ESG performance

With the growing importance of environmental, social, and governance factors in business, investors’ attention to corporate ESG information is increasing, and the inclusion of ESG factors in the investment process has shifted from a niche concept to a mainstream factor [21]. In China, the level of ESG score has become one of the important decision-making factors for investors of market companies. If ESG performance can directly affect the degree of corporate market trading activity, will CEOs actively undertake social responsibility and improve ESG performance due to the pressure from external investors? Research has shown that firms with a high degree of market turnover activity indicate high corporate exposure, and CEOs are likely to use social capital to enhance ESG performance, cater to investor preferences, and create a favorable corporate image due to public pressure [10]. Therefore, a firm’s level of active market trading is likely to play a positive role in the impact of CEO social capital on ESG performance. In addition, ESG-performing firms may receive insurance-like protection and maintain their performance even when facing downside risks [50]. The more active a firm’s market trading is, the stronger the conflict of downside risk is. the CEOs are more in need of ESG performance as a protective umbrella to maintain firm performance. Based on the above analysis, this paper proposes hypothesis H2:

**H2:** Firms’ market trading activity plays a positive moderating role in the effect of CEO social capital on ESG performance.

First, a firm’s board of directors may significantly influence CEO decisions about firm behavior, including ESG performance [51]. Prior research provides evidence that when duality exists (the CEO also serves as the board chair), the CEO’s decisions are free from board interference, less dependent on the board, and conducive to long-term activities, such as ESG practices [27]. Duality strengthens the CEO’s power to react quickly to the external environment and implement innovative actions [52].

In addition, higher-order theory suggests that the personality traits of company executives tend to influence their judgment of themselves and their environment, which is reflected in the company’s decision-making. Research has shown that CEOs who also serve as the chairman of the board have both powers combined and have more power in utilizing internal and external social capital to promote the company’s ESG development [53]. They may adopt some relatively aggressive strategies [54], such as overinvesting and over-indebtedness, and have a greater tolerance for risk and a greater willingness to invest funds in long-term projects. ESG is characterized by long investment horizons, high costs, and uncertain returns, which are compatible with the business style of such CEOs and can be readily values. It is highly likely that CEOs are willing to use their social capital to help companies better fulfill ESG practices and assume social responsibility. Therefore, based on the above analysis, this paper proposes hypothesis H3:

**H3:** CEO duality positively moderates the relationship between CEO social capital and corporate ESG performance.

Firm ownership attributes may also have an impact on the relationship between CEO social capital and firm ESG performance. In China, the government is usually the largest owner of state-owned enterprises (SOEs) and therefore has ultimate control over personnel decisions in these firms, including the selection, appointment, and dismissal of top executives. As a result, SOEs have both economic and political attributes than non-SOEs [55]. On the one hand, as the need for sustainable development is increasingly emphasized, SOEs are likely to be subject to stricter environmental regulatory pressures and market scrutiny [56], and become an important tool for the government to promote high-quality development. CEOs of SOEs are under more pressure to take on ESG or corporate social responsibility activities, and also place more emphasis on ESG performance. Driven by this pressure, CEOs with social capital will be more active in mobilizing resources to enhance corporate ESG performance. On the other hand, thanks to the government’s implicit guarantee, SOEs have fewer financing constraints, more financing channels and lower financing costs [11]. Therefore, ESG practices have a limited impact on the financial position of SOEs. Without the concern of financial burden, CEOs will be more proactive in improving corporate ESG performance and more willing to use their social capital to promote ESG practices. Therefore, based on the above analysis, this paper proposes hypothesis H4:

**H4:** compared to non-state-owned firms, social capital of CEOs of state-owned firms contributes more significantly and positively to firms’ ESG performance.

Firm size is an important factor influencing the relationship between CEO social capital and corporate ESG performance for several possible reasons: First, large-scale firms have entered a period of steady growth, and CEO compensation incentives are relatively homogeneous and mainly linked to firm performance [57]. In order to ensure their own performance, executives are more focused on short-term benefits and are "risk averse" to corporate social responsibility. Small-scale firms have more dynamic CEO compensation systems. The firm is also on the rise and is more interested in long-term sustainability [58]. Second, CEOs of large-scale firms have more centralized power and are also more prone to short-sightedness, avoidance of oversight, and overstepping agency [59]. The social capital of CEOs is more likely to provide the conditions for fraud and collusion, which affect ESG performance. Third, large-scale firms have advantages such as capital, personnel, and technology, and have fewer financing constraints than small-scale firms. However, at the same time, large-scale firms also have problems such as redundancy of institutions and personnel, and lower innovation incentives and innovation efficiency, which may lead to lower ESG performance.

**H5:** Compared to small-scale firms, the negative contribution of social capital of CEOs of large-scale firms to firms’ ESG performance is more significant.

## 3. Research design

### 3.1 Sample selection

In this paper, A-share listed companies in Shanghai and Shenzhen from 2010 to 2020 are selected as samples, and the data of CEO social capital and main financial indicators are from CSMAR database and hand-arranged. In addition, the Hua Zheng ESG rating is adopted as the corporate ESG performance, and the data are from Wind Information Financial Terminal. In order to ensure the validity of the data, the data were screened as follows: (1)the samples of companies with missing CEO data or financial data have been excluded, (2)the samples of companies with missing CSI ESG ratings have been excluded, (3)the samples of ST and ST* companies have been excluded, (4)the samples of financial and insurance companies have been excluded. A total of 27,513 company-year observations were finally obtained. Meanwhile, the first and last 1% shrinkage was applied to all continuous variables.

### 3.2 Main variable definition

From Table 1, we can see the definitions of the main variables.

**Table 1:** Variable definitions.

VarName	Symbol	Variable	Indicators description
Dependent variable	ESG	ESG performance	Company’s Hua Zheng ESG Rating Score
Independent variable	CEO	CEO Social Capital	Based on the above, the aggregate of the six categorized social capitals
Moderator variables	TR	Turnover Rate	Sum of daily turnover (number of shares outstanding) during the year
	duality	CEO duality	CEO duality is a dummy variable (Duality) coded as 1 if the CEO also holds the position of the chairman, and otherwise as 0
Control variables	Lev	The asset-liability ratio	liability/assets
	TobinQ	Company value	Tobin Q value of the company at the end of the period
	Age	Company age	Ln (year of calculation—year of listing + 1)
	Boardsize	Board size	The natural logarithm of the number of board
	Roa	Return on Total Assets	Net profit/average total assets
	Indep	The proportion of Independent Directors	The proportion of independent directors among the board members.
	Year	Year	Year dummy variables by industry
	Ind	Industry	Industry dummy variables by different years

#### Independent variable

Drawing on Helin Sun’s (2023) approach, we use a pooled indicator of CEOs’ embeddedness in the social action network relationships of other stakeholders as a proxy variable for CEO social capital [27]. Specifically, it is equal to the sum of six variables. (1) CEO financial social capital (SC1): the SC is 1 if the CEO has worked in a financial institution such as a bank, and 0 otherwise. (2) Business social capital (SC2): this indicator is measured by the number of firms in which the CEO also serves as a director of other firms. The indicator is based on the mean of the sample, greater than the sample mean SC2 is 1, otherwise 0. (3) Overseas social capital (SC3): 1 if the CEO has worked or studied abroad, otherwise 0. (4) Management social capital: SC4 is 1 if the CEO has pursued an (advanced) MBA or studied at the China Europe Business School (CEBS) or the Cheung Kong Graduate School of Business (CKGSB), otherwise 0. (5) Technology Social Capital (SC5): if the CEO has worked in universities or research institutions, SC5 is 1, otherwise 0. (6) Association Social Capital (SC6): if the CEO has worked in industry associations and business federations, SC6 is 1, otherwise 0.

#### Dependent variable

Considering the applicability period and coverage of each ESG rating, referring to the methodology of Zhou [14], this paper uses Hua Zheng’s ESG rating as a proxy variable for firms’ ESG performance. The Huang Zheng ESG indicator system refers to the mainstream ESG evaluation framework in foreign countries, and combines the reality of China’s capital market and the characteristics of various types of listed companies to construct a three-level indicator system from the top down, which is divided into nine grades, from high to low as AAA, AA, A, BBB, BB, B, CCC, CC, C. For the convenience of analysis, this paper assigns the value of the corporate ESG ratings from the lowest to the highest. When the rating is C take 1, CC take 2 and so on. CSI has been assessing the ESG performance of A-share and debt issuers since 2009, and now it has covered all A-share listed companies, and the index has been widely recognized by the industry and academia.

#### Moderator variables

(1) Turnover Rate, drawing on Itzkowitz, the annual turnover rate of stocks to reflect Market activity level [60]. It is calculated as the sum of the daily turnover rate (number of shares outstanding) during the year, implying the frequency of stock turnover buying and selling in the market during the year. (2) CEO duality, drawing on Helin Sun’s (2023) approach, CEO duality can be determined by checking whether the CEO also serves as chairman of the board of directors. CEO duality is a dummy variable (dichotomous) coded 1 if the CEO also serves as chairman of the board of directors. Otherwise, it is coded 0 [27].

#### Control variables

A firm’s ESG performance can be affected not only by the CEO’s social capital, but also by other firm-level related factors. Drawing on Chen’s approach, this paper also controls for conventional variables affecting firms’ ESG performance in the model, including financial leverage (Lev), firm value (TobinQ), firm’s age at listing (Age), board size (Boardsize), return on assets (Roa), and the percentage of independent directors on the board (Indep) [12].

### 3.3 Model design

This paper focuses on the effect of CEO social capital on corporate ESG. To test hypotheses H1a and H1b, this paper constructs the following benchmark model. The benchmark model can be used to determine the relationship between one or more independent variables and a dependent variable, and it can also help us understand the degree of influence of the independent variable on the dependent variable. (1):

$$ESG=\alpha_{0}+\alpha_{1}{CEO}_{ijt}+\beta_{n}\sum X_{ijt}+\sum D^{Year}+\sum D^{Ind}+\varepsilon_{ijt}$$ (1)

Where subscript i stands for firm, t stands for year, j stands for industry, ∑*X*_*ijt*_ stands for firm-level control variables, ∑*D*^*Year*^ is the year fixed effect, Σ*D*^*Ind*^ is the industry fixed effect, and *ε*_*ijt*_ is the residual term. According to model (1), if the coefficient *α*_*1*_ is significantly negative, it proves that hypothesis H1a is valid. In other words, CEO social capital has a negative impact on corporate ESG performance. Conversely, hypothesis H1b is proved to be valid. In other words, CEO social capital has a positive impact on corporate ESG performance.

We believe that the contribution of CEOs’ social capital to firms’ ESG performance may be affected by some factors, such as the pressure of external investors’ attention and the personality traits of firm executives. When CEOs also hold the position of chairman, they are motivated by performance pressure and their own business philosophy, etc., and are more interested in proving their professional competence to internal and external stakeholders of the firm through ESG performance. Therefore, they may actively utilize their social capital to help improve corporate ESG performance. In addition, firms with high levels of floor trading activity are perceived to be more exposed. CEOs of such firms are likely to pay more attention to corporate ESG performance due to public opinion pressure and investor preferences, and will more actively utilize their social capital to positively influence ESG performance. In order to investigate the mechanism of CEO social capital on corporate ESG and to test hypotheses H2 and H3, this paper adds two moderator variables and their interaction terms with CEO social capital to model (1). These two moderating variables are market trading activity and CEO duality. And, we construct the following models (2)-(3):

$$ESG=\alpha_{0}+\alpha_{1}{CEO}_{ijt}+\alpha_{2}{CEO}_{ijt}\times {TR}_{ijt}+\alpha_{3}{TR}_{ijt}+\beta_{n}\sum X_{ijt}+\sum D^{Year}+\sum D^{Ind}+\varepsilon_{ijt}$$ (2)

$$ESG=\alpha_{0}+\alpha_{1}{CEO}_{ijt}+\alpha_{2}{CEO}_{ijt}\times {duality}_{ijt}+\alpha_{3}{duality}_{ijt}+\beta_{n}\sum X_{ijt}+\sum D^{Year}+\sum D^{Ind}+\varepsilon_{ijt}$$ (3)

## 4. Empirical testing and analysis

### 4.1 Descriptive statistics and analysis

Variables in this paper. As can be seen from the data in the Table 2, the mean ESG score of the sample firms is 6.472 with a standard deviation of 1.106, and the minimum and maximum values are 9.00 and 1.00, which shows that there are large differences in ESG scores between the samples. The mean value of the CEOs is 0.712 with a standard deviation of 0.767, which suggests that most of the firms in the sample do not have a very rich social capital of their CEOs, and that there are differences between the firms are greater. In addition, Table 3 shows that there is no high correlation between the main explanatory variables of the correlation analysis, and the problem of multiple covariates can be excluded.

**Table 2:** Descriptive statistics.

VarName	Obs	Mean	SD	Min	Median	Max
ESG	27513	6.472	1.106	1.000	6.000	9.000
CEO	27513	0.712	0.767	0.000	1.000	3.000
TR	27512	648.526	542.142	55.176	479.334	2806.971
duality	20513	0.271	0.444	0.000	0.000	1.000
Lev	27513	0.423	0.211	0.055	0.413	0.915
TobinQ	27513	2.043	1.285	0.876	1.616	8.036
Age	27513	17.268	5.676	4.000	17.000	31.000
Boardsize	27513	8.587	1.644	5.000	9.000	14.000
Roa	27513	0.039	0.057	-0.197	0.038	0.191
Indep	27513	37.467	5.282	33.330	33.330	57.140

**Table 3:** Pairwise correlations.

Variables	(1)	(2)	(3)	(4)	(5)	(6)	(7)	(8)	(9)	(10)
(1) ESG	1.00									
(2) CEO	-0.05*	1.00								
(3) TR	-0.18*	0.12*	1.00							
(4) duality	-0.10*	0.25*	0.17*	1.00						
(5) Lev	0.09*	-0.13*	-0.22*	-0.15*	1.00					
(6)TobinQ	-0.10*	0.03*	0.11*	0.06*	-0.24*	1.00				
(7) Age	0.06*	-0.07*	-0.16*	-0.10*	0.18*	-0.02	1.00			
(8) Boardsize	0.15*	-0.09*	-0.16*	-0.18*	0.16*	-0.14*	0.02*	1.00		
(9) Roa	0.13*	0.05*	0.06*	0.06*	-0.38*	0.14*	-0.11*	0.00	1.00	
(10) Indep	-0.01	0.04*	0.03*	0.11*	-0.01	0.05*	-0.01	-0.50*	-0.02*	1.00

* show significance at the .01 level

### 4.2 Regression results

This paper uses model (1) to test against hypotheses H1a and H1b, and the regression results are shown in Table 4. Column (1) shows the univariate regression results of CEO social capital on corporate ESG performance, with a regression coefficient of -0.036, showing a negative correlation and significant at the 1% level; the results of adding relevant control variables are shown in column (2), and after controlling for variables that may have an impact on ESG performance, the coefficients of CEO social capital on corporate ESG performance are all improved, with a regression coefficient of -0.027, and remain negatively significant at the 1% level. 0.027 and the results remain negatively significant at the 1% level. The above results support hypothesis H1a and reject hypothesis H1b, and the empirical results show that CEO social capital has a significant negative impact on corporate social responsibility.

**Table 4:** Regression results.

VarName	(1)	(2)
	ESG	ESG
CEO	-0.036***	-0.027***
	(0.008)	(0.008)
Lev		0.328***
		(0.036)
TobinQ		-0.077***
		(0.005)
Age1		0.006***
		(0.001)
Boardsize		0.098***
		(0.005)
Roa1		3.551***
		(0.119)
Indep		0.016***
		(0.001)
_cons	6.498***	4.817***
	(0.009)	(0.082)
Year	Yes	Yes
Ind	Yes	Yes
N	27513	27513
R-squared	0.084	0.137

Standard errors are in parenthesis

*** p<0.01,

** p<0.05,

* p<0.1

### 4.3 Moderating effect test results

The regression results in Table 5 show that: in terms of ESG scores, the coefficient of the interaction term (TRCEO) between CEO social capital and the degree of market turnover activity in column (1) is 0.0001, which is positively significant at the 1% confidence level. This indicates that the degree of market turnover activity plays a positive moderating role in the effect of CEO social capital on ESG performance. Hypothesis H2 is proved; in column (2), the interaction term between CEO social capital and CEO duality (duality CEO) is 0.0689, which is positively significant at 1% confidence level. This finding supports the paper’s hypothesis H3. we argue that the positive contribution of CEO social capital to ESG is more significant when the CEO also holds the position of chairman. In summary the results support the hypotheses H2 and H3 of this paper.

**Table 5:** Moderating effect test.

VarName	(1)	(2)
	ESG	ESG
CEO	-0.0282***	-0.0267***
	(0.0084)	(0.0088)
TRCEO	0.0001***	
	(0.0000)	
TR	-0.0003***	
	(0.0000)	
duality CEO		0.0689***
		(0.0175)
duality		-0.1742***
		(0.0148)
Control variables	Yes	Yes
Year / Ind	Yes	Yes
N	27512	27513
R2	0.086	0.073

Standard errors in parentheses

* *p* < 0.1,

**p < 0.05,

***p < 0.01

### 4.4 Robustness tests

In order to verify the robustness of the previous regression results, we conducted further robustness tests, including Propensity score matching (PSM), Alternative dependent variable and Instrumental variable.

#### 4.4.1 PSM

**Fig 1:**
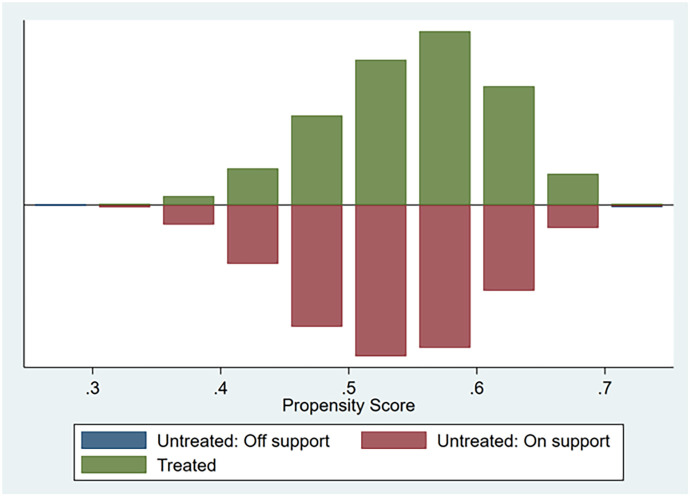
Propensity score matching (PSM).

The relationship between CEO social capital and corporate ESG performance may be affected by the endogeneity problem caused by sample selection. Therefore, in this paper, we use PSM (matching propensity score method) model to mitigate the impact of endogeneity issues on the empirical results. In order to exclude potential selection bias, we match firms whose CEOs have social capital (CEO Social Capital>0) to compare with firms whose CEOs are less social capital (CEO Social Capital = 0). Based on the relevant literature [51], we first use a logit model for estimating the functional relationship between the probability of a firm’s CEO social capital impact and all firm characteristic variables (propensity scores). This also includes firm fixed effects and year fixed effects. Next, based on the predicted probabilities, we classify the probability of being influenced by CEO social capital as being within for 0.005 (absolute value). We match firms whose CEOs have social capital with firms whose CEOs have less social capital. Fig 1 reports the 1:1 matching results. Column (1) of Table 6 reports the post-matching test results. The statistical results show that the effect of CEO social capital on CSR remains significantly negative, supporting hypothesis H1a.

**Table 6:** Robustness tests.

VarName	PSM	Replacement variable	Replacement variable	IV
	(1)	(2)	(3)	(4)
	ESG	NEWESG	SRR	ESG
CEO	-0.32*	-0.031***	-0.015***	-0.071***
	(-1.79)	(0.009)	(0.004)	(0.009)
Control variables	Yes	Yes	Yes	Yes
Year / Ind	Yes	Yes	Yes	Yes
N	27511	23180	23726	27,513
R2	0.148	0.153	0.093	0.002

Standard errors in parentheses

* p<0.1,

** p<0.05,

*** p<0.01

#### 4.4.2 Alternative dependent variable

The choice of variables may have an impact on the empirical results. In order to reduce such errors, robustness tests are conducted here by replacing variables. In the benchmark test, the current ESG score is used as the explanatory variable in this paper. First, we replace the current ESG score with the previous ESG score, and the test results are shown in column (2) of Table 6, the CEO social capital corresponding to NEWESG (previous ESG score) is still significantly negative, and the conclusion remains unchanged. After that, this paper replaces the current ESG score using the current SRR (whether the firm publishes a social responsibility report or not). The test results are shown in column (3) of Table 6, the coefficient of SRR remains significantly negative and the conclusion remains unchanged. Both of the above two replacement variable results prove that the test results of this paper are robust and support hypothesis H1a.

#### 4.4.3 Instrumental variable

We need to consider whether the negative correlation between CEO social capital and firms’ ESG performance may be due to reverse causality? For example, firms with worse ESG performance are more likely to hire CEOs with large social capital. This is because firms with poorer ESG performance have a greater need for CEOs with strong social capital to enhance their firm value. That is, firms’ ESG performance may have an inverse effect on CEO social capital. And, measurement error and omitted variables that have not been included may also lead to endogeneity problems. In this case, the main regression may show a negative correlation between corporate ESG performance and CEO relationship, although the latter has no causal effect on the former.

To alleviate concerns about this type of problem, we refer to Manu (2023) [61] and employ instrumental variable regression. Our instrument is CEO social capital lagged two years. This instrument is used because our results may be affected by the CEO’s current position rather than the CEO’s personal relationships. A valid instrument should be both relevant and exclusive. First, CEO personal resources are continuous, and CEO social capital in the lagged period is necessarily affected by CEO social capital in the current period. It fulfills the instrumental variable relevance requirement. Second, CEO social capital in the lagged period has not yet been realized and is unlikely to have an impact on corporate ESG. Therefore, it satisfies the exclusion condition at the same time.

The first-stage model for the instrumental variable regression is:

$${CEO}_{ijt}=\beta 0\;+\;\beta 1{NEWCEO}_{ijt}+\;\beta 2{Control}_{ijt}+\;INDUSTRY\;+\;YEAR\;+\;{\varepsilon i}_{ijt}$$ (4)

Where *NEWCEO*_*ijt*_ is the CEO’s social capital of the firm lagged two years. Control represents firm- and industry-level control variables. This paper also controls for the fixed effects of *INDUSTRY* and *YEAR*.*εi*_*ijt*_ represents the random error term. The rest of the variables are the same as in model (1). In the first stage regression, the instrument is statistically significant in predicting the CEO connectivity measure. This indicates that it satisfies the correlation requirement. In the second stage of the instrumental variables regression, we use the fitted values of CEO social capital as the explanatory variables to estimate model (4). The results of the two-stage instrumental variable regression are given in Table 6 (4). The results of the second stage regression are consistent with the benchmark results. Therefore, we conclude that CEO social capital leads to a decline in corporate ESG performance.

### 4.5 Further analysis

#### 4.5.1 Grouped by property rights

To explore whether the relationship between CEO social capital and firms’ ESG performance is affected by the nature of property rights, we categorize our sample into state-owned firms (firms with the state as the ultimate controlling party) and non-state-owned firms (other firms as the ultimate controlling party). We then re-execute the regression results. Table 7 reports the results of the conditional effect of CEO social capital on firms’ ESG performance. The univariate regressions of CEO social capital on firms’ ESG performance for firms whose ownership nature is state-owned in columns (1) and (2) show positive correlations with regression coefficients of 0.091 and 0.079, which are significant at the 1% level; Columns (3) (4) show the regression coefficients of CEO social capital on firms’ ESG performance for firms whose ownership nature is non-state. Whether controlling or not, this coefficient is all 0.005, showing significant irrelevance. The results show that the positive contribution of CEO’s social capital to corporate ESG performance is more significant in state-owned enterprises than in non-state-owned enterprises, supporting Hypothesis 4. We believe that state-owned enterprises may be subject to stricter environmental regulatory pressures and market supervision, and CEOs are more pressurized to take on environmental, social, and corporate governance, and also pay more attention to ESG performance. Moreover, SOEs are characterized by fewer financing constraints, more financing channels as well as lower financing costs, and CEOs have fewer financial concerns in utilizing social capital to promote ESG practices.

**Table 7:** Regression results after grouping by nature of ownership.

VarName	(1)	(2)	(3)	(4)
	ESG(State-owned)	ESG(State-owned)	ESG(Non-State Owned)	ESG(Non-State Owned)
CEO	0.091***	0.079***	0.005	0.005
	(0.018)	(0.017)	(0.009)	(0.009)
Control variables	No	Yes	No	Yes
Year / Ind	Yes	Yes	Yes	Yes
N	9,549	9,549	17,095	17,095
R2	0.068	0.068	0.061	0.061

Standard errors are in parenthesis

*** p<0.01,

** p<0.05,

* p<0.1

#### 4.5.2 Grouped by size

To explore whether the relationship between CEO social capital and firms’ ESG performance is affected by firm size, we take the median of the Size variable (ln(1+ Total Assets)) and divide the sample into small-sized firms (Size variable less than the median) and large-sized firms (Size variable greater than or equal to the median). Then, we re-execute the regression results. Table 8 reports the results of the conditional effect of CEO social capital on firms’ ESG performance. Column (1) is a univariate regression of CEO social capital on firms’ ESG performance under the large-scale firms sample. The regression coefficient is -0.066, which shows negative correlation and is significant at the 1% level; column (2) shows a regression result of 0.015, which is significantly uncorrelated, of CEO social capital on corporate ESG performance under the sample of small-scale firms. The results show that: compared to small-scale firms, the negative contribution of CEO’s social capital of large-scale firms to firms’ ESG performance is more significant. Hypothesis 5 is supported. We believe that there are many problems in large-scale firms, such as a single compensation incentive system, more centralized power of CEOs, lower innovation motivation and innovation efficiency, and so on, which lead to a significant negative relationship between CEO social capital and ESG performance.

**Table 8:** Regression results after grouping by firm size.

VarName	(1)	(2)
	ESG(Large-scale)	ESG(Small-scale)
CEO	-0.066***	0.015
	(0.014)	(0.010)
Control variables	Yes	Yes
Year / Ind	Yes	Yes
N	12182	15331
R2	0.122	0.107

Standard errors are in parenthesis

*** p<0.01,

** p<0.05,

* p<0.1

### 4.6 CEO social capital, ESG performance and EPS

Although corporate ESG practices satisfy the interests of some stakeholders, such as bringing a good social reputation to the CEO and the company, enhancing shareholder trust, improving the efficiency of corporate financing, increasing customer and supplier satisfaction with the company, increasing sales revenue, and reducing transaction costs. However, the expense costs paid by enterprises for ESG practices, such as purchasing environmental protection equipment and improving the production environment, also increase the financial burden to a certain extent. Therefore, it is necessary to further discuss whether corporate ESG practices bring wealth increase to shareholders. Therefore, in this paper, EPS (Profit after tax/Total share capital) is used as the explained variable, and the cross-multiplier of CEO’s social capital with ESG score (E-CEO) is included in the model as the explained variable in focus. The results, as shown in Table 9, show that the coefficient of E- CEO is negatively significant at the 1% level. This indicates that ESG practices under the influence of CEO social capital reduce shareholder wealth.

**Table 9:** CEO social capital, ESG performance and EPS.

VarName	EPS
ESG	0.045***
	(0.003)
CEO	0.045**
	(0.019)
E- CEO	-0.007***
	(0.003)
Control variables	Yes
Year / Ind	Yes
N	6235
R2	0.254

Standard errors are in parenthesis

*** p<0.01,

** p<0.05,

* p<0.1

## 5. Conclusion

In recent years, as the need for sustainable development has been increasingly emphasized, companies with excellent ESG performance are more likely to receive external attention and praise. The influencing factors of ESG include external factors, such as the attention of society, government, investors, and the media. At the same time, it also includes internal factors, such as corporate values, management traits, and shareholder bias, etc. The CEO’s personal social capital, as a management trait, is an important internal factor. Does it have a significant impact on corporate ESG performance? Does this impact reflect heterogeneity with different corporate characteristics? This paper empirically examines the relationship between CEO social capital and corporate ESG performance using a sample of A-share listed companies in Shanghai and Shenzhen from 2010 to 2020. It is found that CEO social capital and corporate ESG performance are negatively correlated, and CEO social capital leads to a decline in corporate ESG performance. Our findings deepen the knowledge of academics and practitioners about the value-creating function of CEO social capital. At the same time, it provides a new basis for listed companies to emphasize and utilize CEO social capital to enhance corporate social responsibility undertaking.

In addition, we consider how a firm’s market turnover and CEO duality moderates the impact of CEO social capital on a firm’s ESG. When the degree of market turnover is higher, which means that investors are more concerned about the firm, CEOs may use their social capital more actively to help firms improve their ESG performance due to public pressure and performance impact. We have tested this by moderating effects. The results show that the firm’s market trading activity positively moderates the relationship between CEO social capital and firm’s ESG performance. Higher-order theory suggests that the personality traits of corporate executives tend to influence their judgment of themselves and their environment, which is reflected in the firm’s decision-making. CEOs who also serve as chairman of the board tend to have greater corporate dominance, are less dependent on the board of directors, and are able to invest in long-term programs such as ESG. We have demonstrated through moderating effects tests that CEO duality positively moderates the relationship between CEO social capital and corporate ESG performance.

Next, we further categorize the sample into SOEs and non-SOEs, large-scale firms and other firms. We test the moderating effects of property rights nature and size on CEO social capital on firms’ ESG performance. State-owned enterprises (SOEs) are characterized by fewer financing constraints, lower financing costs, and higher environmental regulatory pressures. Therefore, CEOs of SOEs have a greater need to reflect their performance through ESG performance. The empirical results also show that the positive contribution of CEO’s social capital to corporate ESG performance is more significant in state-owned enterprises. Compared to the flexible mechanism of small-scale enterprises, large-scale enterprises have the problems of single compensation incentive system, lower innovation motivation and innovation efficiency. After our study, the results show that the negative relationship between CEO social capital and ESG performance of large-scale enterprises is more significant.

Finally, we consider that while corporate ESG practices bring good social reputation to the CEO and the company. But it also increases the corporate financial burden. According to the theory of shareholder wealth maximization, do corporate ESG practices bring wealth increase to shareholders? After the experiment, we found that ESG practices decrease shareholder wealth under the influence of CEO’s social capital.
